# Trajectories of adiposity indices and the risk of cardiovascular disease and mortality: a prospective cohort study

**DOI:** 10.1186/s12967-025-07467-2

**Published:** 2026-01-21

**Authors:** Mahdieh Golzarand, Maryam Mahdavi, Behnaz Abiri, Parvin Mirmiran, Fereidoun Azizi

**Affiliations:** 1 Nutrition and Endocrine Research Center, Research Institute for Endocrine Sciences, Shahid Beheshti University of Medical Sciences, No. 23, Shahid Arabi St., Yemen St., Chamran Exp., Tehran, 193954763 Iran; 2 Obesity Research Center, Research Institute for Endocrine Sciences, Shahid Beheshti University of Medical Sciences, Tehran, Iran; 3 Department of Clinical Nutrition and Dietetics, Faculty of Nutrition Sciences and Food Technology, National Nutrition and Food Technology Research Institute, Shahid Beheshti University of Medical Sciences, No.47, Shahid Hafezi St., Farahzadi Blvd. Sharak-e-Ghods, Tehran, 1981619573 Iran; 4 Endocrine Research Center, Research Institute for Endocrine Sciences, Shahid Beheshti University of Medical Sciences, Tehran, Iran

**Keywords:** Cardiovascular events, Mortality, Body roundness index, Adiposity, Body shape index, Cancer

## Abstract

**Background:**

While novel adiposity indices such as the body roundness index (BRI), a body shape index (ABSI), the visceral adiposity index (VAI), and the Clínica Universidad de Navarra-Body Adiposity Estimator (CUN-BAE) index outperform body mass index (BMI) in predicting cardiovascular disease (CVD) risk, their long-term trajectories remain unstudied in Iran. We investigated the patterns of the BRI/ABSI/VAI/CUN-BAE index and their associations with the risk of CVD, all-cause mortality, and specific-cause mortality among Iranian adults.

**Methods:**

This prospective cohort study analyzed 11,394 Iranian adults (55.5% women, mean age 41.2 ± 15.0 years) from the Tehran Lipid and Glucose Study (1999–2018). Latent class growth mixture modeling identified trajectories of adiposity indices over a median of 18.0 years. Cox models assessed associations between the trajectories and incident CVD (*n* = 728), all-cause mortality (*n* = 532), and cause-specific mortality, adjusting for various socio-demographic, lifestyle, and metabolic confounders.

**Results:**

Three distinct trajectories (low, moderate, and high-increase) emerged for all indices, with the high-increasing trajectories showing the strongest associations for CVD risk, the CUN-BAE index (HR: 2.45, 95%CI: 1.73–3.49), BRI (HR: 2.12, 95%CI: 1.65–2.73), ABSI (HR: 2.02, 95%CI: 1.38–2.95), and VAI (HR: 1.92, 95% CI: 1.49–2.49). Besides, the high-increase in BRI was associated with a higher risk of all-cause mortality (HR: 1.47, 95% CI: 1.10–1.96) and cardiovascular mortality (HR: 3.25, 95% CI: 1.85–5.69) compared with the low-increase group. There was no relationship between trajectory in the CUN-BAE index, VAI, and ABSI and risk of all-cause and cause-specific mortality. Notably, no significant associations were observed between any adiposity index trajectories and cancer mortality.

**Conclusion:**

Longitudinal trajectories of adiposity indices particularly BRI strongly predict CVD and mortality risks in Iranian adults. These findings support the incorporation of dynamic adiposity measures into clinical risk stratification.

**Supplementary Information:**

The online version contains supplementary material available at 10.1186/s12967-025-07467-2.

## Introduction

Cardiovascular disease (CVD) is a major contributor to disabilities and mortality worldwide [1], and obesity is one of the primary causes of CVD, which imposes significant costs on communities [2]. The World Health Organization (WHO) report indicates that, over the past three decades, the prevalence of obesity, defined by body mass index (BMI), has doubled among adults worldwide [3]. However, it cannot differentiate between body fat mass (FM) and lean body mass (LBM) [4].

Evidence demonstrated that adiposity, particularly abdominal adiposity, is a better predictor of health risks than obesity [5, 6]. Few accurate techniques exist for measuring adiposity, such as computed tomography (CT) and dual-energy X-ray absorptiometry (DXA). Still, their high cost restricts their application in epidemiological studies [7]. Therefore, there is a need for low-cost yet highly accurate alternative methods. Waist circumference (WC) and the WC-to-height ratio (WHtR) are two recommended proxies for abdominal adiposity; nevertheless, they cannot distinguish between subcutaneous and visceral adiposity [8, 9]. In recent years, several novel anthropometric indices have been introduced as an alternative to the classic anthropometric indices, including the body roundness index (BRI), a body shape index (ABSI), the Clínica Universidad de Navarra-Body Adiposity Estimator (CUN-BAE) index, and the visceral adiposity index (VAI). In 2013, BRI was introduced to predict percent body fat and visceral adiposity by applying WC and height [10]. ABSI was developed in 2012 by combining WC, weight, and height to measure abdominal adiposity [11]. VAI is a sex-specific tool that estimates visceral adiposity based on WC, height, triglyceride, and high-density cholesterol (HDL) [12]. The CUN-BAE index was developed by incorporating age, sex, and BMI into a matrix to determine percent body fat [13].

Previous documents have indicated the WHtR is a strong predictor of CVD and mortality in the Iranian population [14–18]. In contrast, few studies have focused on these novel indices and established their association with the incidence of CVD and mortality [19–23]. Additionally, most previous studies used a single point in time for the anthropometric index. Since these indices can change over time and the trend of changes may vary between individuals, using the trajectories of indices may represent a more accurate association with outcomes. Currently, no studies have examined the trajectories of adiposity indices with incident CVD or mortality within the Iranian population. The predictive value of these indices has not been previously assessed in this population. Therefore, the present longitudinal research aimed to identify the association between the BRI, ABSI, VAI, and CUN-BAE index trajectories and the risk of CVD, all-cause and specific-cause mortality, and compare their predictive abilities among Iranian adults.

## Methods

### Study population

This cohort study applied the Tehran Lipid and Glucose Study (TLGS) data [24]. We included 12,981 individuals aged 19 years or older who enrolled in the first phase of TLGS (1999–2001) and new participants who enrolled in the second phase of TLGS (2002–2005). After excluding individuals with incomplete data at baseline (*n* = 511) and those who missed the follow-up time point or had missing data during the follow-up (*n* = 1,076), a total of 11,394 eligible participants were included in this study and were followed until 2018. To evaluate CVD risk, we also removed participants who had no data about previous cardiovascular events at baseline (*n* = 2,172) as well as those with a history of CVD at baseline (*n* = 500). Finally, 8,722 individuals were included in the study, and their trajectory models for each outcome were identified.

We conducted the present study by the Helsinki Declaration of Ethics. The study protocol was approved by the ethics committee of Shahid Beheshti University of Medical Sciences, and written consent was obtained from all participants (IR.SBMU.ENDOCRINE.REC.1404.011).

### Participants’ data

At baseline, all participants were invited to the TLGS unit, and the following information was collected via in-person interview: socio-demographic factors, physical examination results, lifestyle data, and medical histories. The participants’ data were recollected every three years, at specific intervals such as 2002–2005, 2006–2008, 2009–2011, 2012–2014, and 2015–2018, to update the prospective TLGS database. The physical activity of participants was assessed using two different methods. In the first phase of TLGS, the Lipid Research Clinics (LRC) questionnaire was utilized [25]. However, since the second phase of TLGS, the validated Modifiable Activity Questionnaire (MAQ) [26] has been used instead of the LRC questionnaire, as it yields more accurate results for the Iranian culture. We classified the total activity score into two categories: low activity (less than 600 MET minutes per week) and moderate to high activity (more than 600 MET minutes per week). Laboratory tests, including fasting serum glucose and lipid profiles, were performed after a fasting period of 10 to 12 h using an enzymatic colorimetric assay. Dietary intake was evaluated through two 24-hour dietary recalls during both phases I and II. A random subsample of our population, consisting of 1,476 individuals, was selected to participate in face-to-face interviews to gather dietary data. In this study, we were unable to assess alcohol consumption patterns or the amounts consumed, as alcohol intake is prohibited for religious reasons in Iran.

### Measurement of adiposity indices

Body weight was measured using a digital scale (Seca 707, Germany), height was measured using a fixed tape measure, and WC was measured using a flexible tape measure near the umbilicus. The BMI was calculated by dividing weight (kg) by height squared (m). We calculated the adiposity indices, including the BRI [10], ABSI [11], VAI [12], and CUN-BAE index [13], using the proposed equations as represented in Supplementary Table 1.

### Ascertainment of outcomes

In this study, a qualified nurse annually assessed cardiovascular events, all-cause deaths, and cause-specific deaths by telephone. Upon reporting an event, a professional physician collected the participants’ medical records or death certificates for subsequent evaluation by the TLGS outcome committee. The 10th edition of the International Classification of Diseases (ICD-10) was used to diagnose cardiovascular events. These included definite and probable myocardial infarction (MI), unstable angina, coronary heart disease (CHD) that was shown by an angiography, heart failure, CHD death, stroke, or a transitory ischemic event [27].

### Statistical analysis

The baseline characteristics of participants are presented as percentages for categorical variables and the means with standard deviation (SD) for continuous variables. To compare these traits based on the occurrence of the outcomes, we conducted an analysis of variance (ANOVA) and a Chi-squared test as appropriate. The duration of follow-up was defined from the baseline until December 31, 2018, the date of outcome occurred, or the last date of examination, whichever appeared first. The Latent Class Growth Analyses (LCGA) is a semi-parametric method used to identify distinct subgroups of individuals following similar patterns of change over time in a given variable. In this analysis, this approach identifies patterns of adiposity indices, which classify individuals with similar behavioral trajectories. Model selection was conducted in two steps. The number of trajectory groups was determined based on the Bayesian information criterion (BIC) and their substantive significance. In the second step, we tested different shapes for each latent class to identify the pattern of change over time (linear, quadratic, or cubic). In all analyses, minimum class sizes of at least 5% of the sample were considered. Based on the graphic patterns of the trajectories, we labeled them (Supplementary Fig. 1). To calculate the annual growth rate for each adiposity index, the changes between the final measurement and the baseline measurement were computed and then divided by the year between measurements. The ANOVA was used to compare the annual growth rate between groups. The Cox proportional hazards models were performed to assess the association between trajectories in adiposity indices and the risk of CVD, all-cause mortality, and specific cause mortality. We reported the results as a hazard ratio (HR) and 95% confidence interval (CI) for each outcome of interest. For CVD, Model 1 was adjusted for sex (men or women) and age (year). Model 2 was adjusted for Model 1, plus smoking (yes or no), physical activity level (low or high), education level (illiterate, undergraduate, or graduate), and use of antihyperlipidemic medications (yes or no). Model 3 was adjusted for Model 2, plus a history of type 2 diabetes (yes or no) and hypertension (yes or no). For mortality, Model 1 was adjusted for sex (men or women) and age (year). Model 2 was adjusted for Model 1, plus smoking (yes or no), physical activity level (low or high), education level (illiterate, undergraduate, or graduate), and use of antihyperlipidemic medications (yes or no). Model 3 was adjusted for Model 2, plus history of type 2 diabetes (yes or no), hypertension (yes or no), CVD (yes or no), and cancer (yes or no). Hypertension was defined as systolic blood pressure ≥ 130 mmHg or diastolic blood pressure ≥ 85 mmHg, or the use of antihypertensive medications. Fasting serum glucose ≥ 100 mg/dL or taking antidiabetic medication was considered type 2 diabetes. In this study, due to the limited number of participants in the population’s dietary intake and the lack of data regarding alcohol consumption, we did not adjust the final model based on these factors. The model’s proportional hazards (PH) assumptions were assessed with Schoenfeld residuals. We used Harrell’s concordance index (C-index) to assess the model’s discrimination power. Sensitivity analysis was performed by removing participants with an age under 30 years and a history of diabetes or hypertension at baseline. Some studies have indicated that the effect of obesity on CVD is cumulative and depends on the duration of obesity. In this cohort, we found no outcome events within the first two years of follow-up periods, so the sensitivity analysis based on this factor was not performed. For competing risk data, we conducted Fine-Gray model analysis, considering non-cardiovascular mortality as a competing risk event. All data were analyzed using SPSS software (version 20.0; IBM Corporation, Armonk, NY, USA) and Stata. A two-tailed P-value less than 0.05 was considered significant.

## Results

**Fig. 1:**
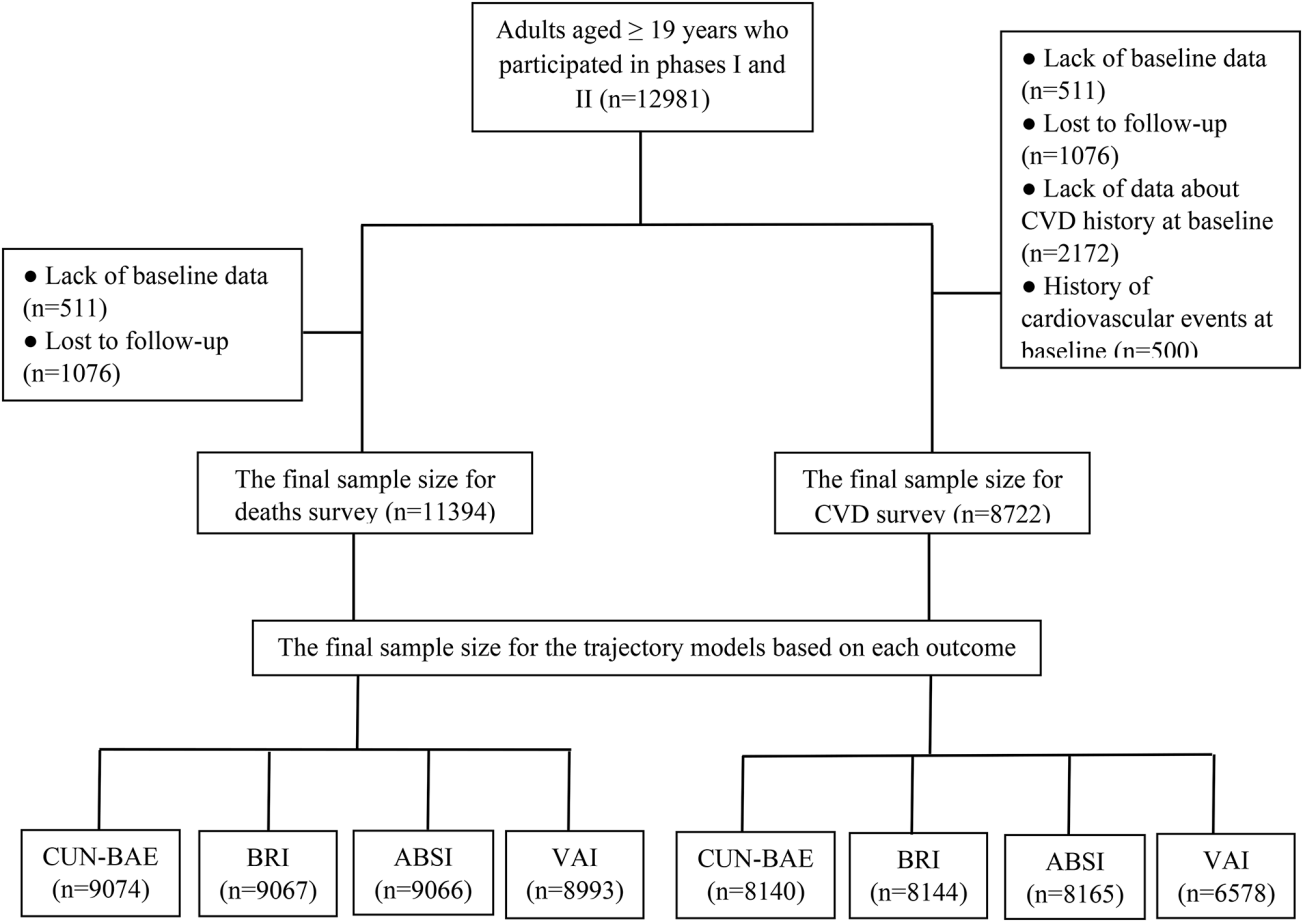
The flow chart of study

In this study, 11,394 men and women participated in evaluating mortality, and 8,722 were included for CVD. The final sample sizes for the trajectory models based on each outcome are presented in Fig. 1. During a median of 18.0 years of follow-up, 728 cases of CVD, 532 cases of all-cause deaths, 144 CVD-related deaths, and 116 cases of cancer-related deaths were recorded. The mean age of the participants was 41.2 ± 15.0 years, and their BMI was 26.7 ± 4.7 kg/m². A total of 55.5% of participants were women, and 4.5%, 10.5%, and 20.7% of those suffered from CVD, type 2 diabetes, and hypertension, respectively.

We identified the best-fitting model among our participants, which was the model with three adiposity index trajectories: low-increase, moderate-increase, and high-increase (Supplementary Fig. 1). The baseline characteristics of participants according to the CUN-BAE index trajectory, which has the largest sample size, are presented in Table 1. Table 2 represents the annual growth rate and the distribution of low-, moderate-, and high-increase in each adiposity index based on the occurrence of CVD.

**Table 1:** Baseline characteristics of participants based on the CUN-BAE index trajectory

General characteristics	Low-increase (*n* = 2825)	Moderate-increase (*n* = 3129)	High-increase (*n* = 3120)	*P* value
Age (year)	38.1 ± 14.2	38.5 ± 14.1	44.6 ± 12.5	< 0.0001
Women (%)	209 (7.40)	1829 (58.5)	3073 (98.5)	< 0.0001
Weight (kg)	67.5 ± 10.7	69.4 ± 15.2	73.8 ± 11.5	< 0.0001
BMI (kg/m^2^)	23.4 ± 3.07	26.0 ± 3.86	30.4 ± 3.40	< 0.0001
Waist (cm)	82.6 ± 9.71	86.7 ± 12.9	94.1 ± 10.7	< 0.0001
The CUN-BAE index	21.3 ± 5.09	31.4 ± 3.95	42.4 ± 4.36	< 0.0001
Body roundness index	3.17 ± 1.01	4.06 ± 1.34	5.61 ± 1.63	< 0.0001
A body shape index	0.077 ± 0.005	0.077 ± 0.006	0.078 ± 0.006	0.042
Visceral adiposity index	2.65 ± 2.96	2.90 ± 2.71	3.77 ± 2.89	< 0.0001
Type 2 diabetes (%)	161 (6.60)	256 (9.30)	393 (13.6)	< 0.0001
Hypertension (%)	322 (11.8)	497 (16.7)	868 (28.7)	< 0.0001
Smoking (%)	705 (25.5)	326 (10.6)	102 (3.30)	< 0.0001
Low physical activity (%)	1890 (68.5)	2129 (69.4)	2125 (68.9)	0.770
Education				< 0.0001
*Illiterate (%)*	65 (2.30)	141 (4.50)	437 (14.0)	
*Up to diploma (%)*	2206 (78.1)	2521 (80.6)	2484 (79.6)	
*Graduated (%)*	554 (19.6)	466 (14.9)	199 (6.40)	
FSG (mg/dL)	93.2 ± 23.3	94.7 ± 27.2	100 ± 35.1	< 0.0001
Total cholesterol (mg/dL)	192 ± 42.8	200 ± 43.9	220 ± 47.3	< 0.0001
Triglyceride (mg/dL)	160 ± 118	160 ± 120	180 ± 108	< 0.0001
HDL cholesterol (mg/dL)	39.2 ± 9.92	42.3 ± 11.2	43.7 ± 11.0	< 0.0001
LDL cholesterol (mg/dL)	122 ± 35.5	127 ± 36.8	141 ± 38.9	< 0.0001
SBP (mmHg)	115 ± 15.0	116 ± 18.0	122 ± 19.4	< 0.0001
DBP (mmHg)	74.9 ± 10.0	76.0 ± 10.7	79.7 ± 10.4	< 0.0001

Data are presented as frequency (percentages) for categorical variables and mean (SD) for continuous variables

To compare data, the ANOVA and Chi-squared were used as appropriate

BMI, body mass index; DBP, diastolic blood pressure; FSG, fasting serum glucose; HDL, high-density lipoprotein; LDL, low-density lipoprotein; SBP, systolic blood pressure

**Table 2:** Annual growth rate and distribution of low-, moderate-, and high-increase of adiposity indices stratified by cardiovascular disease

Trajectories of adiposity indices	Annual growth rate	Distribution		*P* value
		Yes (*n* = 3129)	No (*n* = 3120)	
**The CUN-BAE index**				
Low-increase	0.301 ± 0.005	189 (7.60)	2311 (92.4)	< 0.0001
Moderate-increase	0.282 ± 0.005	240 (8.50)	2588 (91.5)	
High-increase	0.213 ± 0.005^*^	299 (10.6)	2513 (89.4)	
**Body roundness index (BRI)**				
Low-increase	0.065 ± 0.001	166 (4.40)	3581 (95.6)	< 0.0001
Moderate-increase	0.076 ± 0.001	382 (11.4)	2958 (88.6)	
High-increase	0.112 ± 0.002^*^	180 (17.3)	861 (82.7)	
**A body shape index (ABSI)**				
Low-increase	0.00027 ± 0.000	38 (1.90)	1969 (98.1)	< 0.0001
Moderate-increase	0.00024 ± 0.000	355 (8.10)	4032 (91.9)	
High-increase	0.00020 ± 0.000^*^	335 (19.2)	1410 (80.8)	
**Visceral adiposity index (VAI)**				
Low-increase	0.008 ± 0.004	96 (4.90)	1855 (95.1)	< 0.0001
Moderate-increase	-0.027 ± 0.003	365 (11.1)	2920 (88.9)	
High-increase	-0.074 ± 0.005^*^	196 (14.9)	1120 (85.1)	

Data are presented as frequency (percentages) for categorical variables and mean (SD) for continuous variables

The ANOVA was used to compare the annual growth rate between groups

The Chi-squared was used to compare the distribution of the outcome

^*^P-value < 0.0001

Table 3 represents the HRs (95% CI) for CVD by trajectories in the CUN-BAE index, BRI, ABSI, and VAI. The multivariate adjusted HRs for CVD were 1.36 (95% CI: 1.10–1.67) and 2.45 (95% CI: 1.73–3.49) respectively for the moderate-increase and the high-increase in CUN-BAE index when compared to the low-increase in CUN-BAE index; 1.77 (95% CI: 1.46–2.15) and 2.12 (95% CI: 1.65–2.73) for the moderate-increase and the high-increase in BRI in comparison with the low-increase in BRI; 2.02 (95% CI: 1.42–2.88) and 2.02 (95% CI: 1.38–2.95) for the moderate-increase and the high-increase in ABSI compared with the low-increase in ABSI; 1.58 (95% CI: 1.25–1.99) and 1.92 (95% CI: 1.49–2.49) for the moderate-increase and the high-increase in VAI compared with the low-increase in VAI. The C-indexes were 0.812 (95% CI: 0.798–0.825) for the CUN-BAE index; 0.814 (95% CI: 0.800-0.827) for BRI, 0.810 (95% CI: 0.796–0.824) for ABSI; and 0.805 (95% CI: 0.791–0.820) for VAI. The PH assumption was not violated for the CUN-BAE index (*P* = 0.50), BRI (*P* = 0.53), ABSI (*P* = 0.33), and VAI (*P* = 0.66).

**Table 3:** Hazard ratio (95% CI) for the incidence of cardiovascular disease by trajectories in adiposity indices

The CUN-BAE index	no/total	Crude model	Model 1⸶	Model 2⸷	Model 3†
Low-increase	189/2500	1	1	1	1
Moderate-increase	240/2828	1.12 (0.92–1.36)	1.54 (1.26–1.89)^⁑^	1.57 (1.28–1.93)^⁑^	1.36 (1.10–1.67)^*^
High-increase	299/2812	1.31 (1.08–1.58)^*^	3.12 (2.21–4.41)^⁑^	3.12 (2.20–4.42)^⁑^	2.45 (1.73–3.49)^⁑^
**Body roundness index (BRI)**		**Crude model**	**Model 1**⸶	**Model 2**⸷	**Model 3**†
Low-increase	168/3593	1	1	1	1
Moderate-increase	382/2960	2.56 (2.12–3.09)^⁑^	1.92 (1.58–2.33)^⁑^	1.93 (1.59–2.35)^⁑^	1.77 (1.46–2.15)^⁑^
High-increase	180/861	3.94 (3.17–4.90)^⁑^	2.72 (2.13–3.47)^⁑^	2.63 (2.06–3.37)^⁑^	2.12 (1.65–2.73)^⁑^
**A body shape index (ABSI)**		**Crude model**	**Model 1**⸶	**Model 2**⸷	**Model 3**†
Low-increase	38/1974	1	1	1	1
Moderate-increase	357/4048	4.25 (3.01–5.99)^⁑^	2.26 (1.59–3.22)^⁑^	2.18 (1.53–3.10)^⁑^	2.02 (1.42–2.88)^⁑^
High-increase	336/1412	10.78 (7.63–15.21)^⁑^	2.49 (1.71–3.62)^⁑^	2.35 (1.61–3.43)^⁑^	2.02 (1.38–2.95)^⁑^
**Visceral adiposity index (VAI)**		**Crude model**	**Model 1**⸶	**Model 2**⸷	**Model 3**†
Low-increase	96/1960	1	1	1	1
Moderate-increase	368/3297	2.18 (1.74–2.74)^⁑^	1.79 (1.43–2.25)^⁑^	1.80 (1.43–2.26)^⁑^	1.58 (1.25–1.99)^⁑^
High-increase	196/1321	2.98 (2.33–3.81)^⁑^	2.52 (1.96–3.23)^⁑^	2.47 (1.92–3.17)^⁑^	1.92 (1.49–2.49)^⁑^

The Cox proportional hazards models were used

⸶Model 1 adjusted for sex (men or women) and age (year)

⸷Model 2 adjusted for sex (men or women), age (year), smoking (yes or no), physical activity level (low or high), education (illiterate, undergraduate, or graduate), and antihyperlipidemic medications (yes or no)

†Model 3 adjusted for sex (men or women), age (year), smoking (yes or no), physical activity level (low or high), education (illiterate, undergraduate, or graduate), antihyperlipidemic medications (yes or no), and history of type 2 diabetes (yes or no) and hypertension (yes or no)

^*^P-value < 0.05

^⁑^P-value < 0.0001

Tables 4, 5 and 6 represent the estimated risk for all-cause and specific cause mortality across the CUN-BAE index, BRI, ABSI, and VAI trajectories. In the fully adjusted model, the HR (95% CI) for all-cause mortality was 1.47 (95% CI: 1.10–1.96) for the high-increase in BRI compared with the low-increase (Model 3, Table 4). In contrast, no association was found between the trajectory in the CUN-BAE index, ABSI, and VAI and the risk of all-cause mortality in the final model (Table 4). The C-indexes were 0.865 (95% CI: 0.848–0.881) for CUN-BAE index, 0.866 (95% CI: 0.850–0.883) for BRI, 0.865 (95% CI: 0.849–0.882) for ABSI, and 0.865 (95% CI: 0.848–0.881) for VAI. The PH assumption was not violated for the CUN-BAE index (*P* = 0.46), BRI (*P* = 0.41), ABSI (*P* = 0.38), and VAI (*P* = 0.35).

**Table 4:** Hazard ratio (95% CI) for the incidence of all-cause mortality by trajectories in adiposity indices

The CUN-BAE index	no/total	Crude model	Model 1⸶	Model 2⸷	Model 3†
Low-increase	151/2674	1	1	1	1
Moderate-increase	191/2938	1.12 (0.90–1.40)	1.18 (0.93–1.49)	1.19 (0.94–1.50)	1.08 (0.85–1.37)
High-increase	190/2930	1.03 (0.82–1.29)	1.10 (0.73–1.67)	1.05 (0.69–1.60)	0.89 (0.58–1.36)
**Body roundness index (BRI)**		**Crude model**	**Model 1**⸶	**Model 2**⸷	**Model 3**†
Low-increase	148/4040	1	1	1	1
Moderate-increase	240/3465	1.69 (1.36–2.09)^⁑^	1.10 (0.89–1.37)	1.09 (0.87–1.36)	1.00 (0.80–1.24)
High-increase	142/1032	3.24 (2.55–4.12)^⁑^	1.85 (1.41–2.43)^⁑^	1.74 (1.32–2.30)^⁑^	1.47 (1.10–1.96)^*^
**A body shape index (ABSI)**		**Crude model**	**Model 1**⸶	**Model 2**⸷	**Model 3**†
Low-increase	21/2137	1	1	1	1
Moderate-increase	183/4631	4.11 (2.53–6.67)^⁑^	1.42 (0.86–2.33)	1.35 (0.82–2.22)	1.21 (0.74–1.99)
High-increase	326/1768	16.58 (10.30–26.70)^⁑^	1.71 (1.03–2.85)^*^	1.58 (0.95–2.63)	1.36 (0.82–2.26)
**Visceral adiposity index (VAI)**		**Crude model**	**Model 1**⸶	**Model 2**⸷	**Model 3**†
Low-increase	98/2597	1	1	1	1
Moderate-increase	292/4638	1.52 (1.20–1.93)^⁑^	1.32 (1.04–1.67)^*^	1.30 (1.02–1.65)^⁑^	1.16 (0.91–1.47)
High-increase	130/1758	1.73 (1.32–2.27)^⁑^	1.61 (1.22–2.13)^*^	1.55 (1.17–2.05)^⁑^	1.26 (0.94–1.69)

The Cox proportional hazards models were used

⸶Model 1 adjusted for sex (men or women) and age (year)

⸷Model 2 adjusted for sex (men or women), age (year), smoking (yes or no), physical activity level (low or high), education (illiterate, undergraduate, or graduate), and antihyperlipidemic medications (yes or no)

†Model 3 adjusted for sex (men or women), age (year), smoking (yes or no), physical activity level (low or high), education (illiterate, undergraduate, or graduate), antihyperlipidemic medications (yes or no), and history of type 2 diabetes (yes or no), hypertension (yes or no), CVD (yes or no) and cancer (yes or no)

^*^P-value < 0.05

^⁑^P-value < 0.0001

**Table 6:** Hazard ratio (95% CI) for the incidence of cancer mortality by trajectories in adiposity indices

The CUN-BAE index	no/total	Crude model	Model 1⸶	Model 2⸷	Model 3†
Low-increase	38/2787	1	1	1	1
Moderate-increase	43/3086	1.12 (0.71–1.76)	1.06 (0.65–1.74)	1.07 (0.65–1.77)	1.10 (0.67–1.82)
High-increase	35/3085	0.79 (0.49–1.28)	0.55 (0.24–1.22)	0.54 (0.24–1.22)	0.54 (0.24–1.23)
**Body roundness index (BRI)**		**Crude model**	**Model 1**⸶	**Model 2**⸷	**Model 3**†
Low-increase	44/4144	1	1	1	1
Moderate-increase	50/3655	1.20 (0.79–1.83)	0.77 (0.50–1.20)	0.77 (0.50–1.20)	0.78 (0.50–1.22)
High-increase	21/1153	1.69 (1.00-2.86)	0.89 (0.49–1.63)	0.89 (0.49–1.63)	0.91 (0.49–1.69)
**A body shape index (ABSI)**		**Crude model**	**Model 1**⸶	**Model 2**⸷	**Model 3**†
Low-increase	7/2053	1	1	1	1
Moderate-increase	43/4663	2.84 (1.21–6.70)^*^	1.10 (0.46–2.68)	1.09 (0.45–2.65)	1.10 (0.45–2.70)
High-increase	65/2235	8.76 (3.78–20.21)^⁑^	1.16 (0.46–2.93)	1.15 (0.46–2.90)	1.18 (0.46-3.00)
**Visceral adiposity index (VAI)**		**Crude model**	**Model 1**⸶	**Model 2**⸷	**Model 3**†
Low-increase	24/2597	1	1	1	1
Moderate-increase	65/4638	1.45 (0.89–2.36)	1.25 (0.77–2.05)	1.23 (0.76–2.04)	1.30 (0.79–2.13)
High-increase	24/1758	1.41 (0.79–2.53)	1.29 (0.71–2.35)	1.28 (0.70–2.34)	1.39 (0.76–2.56)

The Cox proportional hazards models were used

⸶Model 1 adjusted for sex (men or women) and age (year)

⸷Model 2 adjusted for sex (men or women), age (year), smoking (yes or no), physical activity level (low or high), education (illiterate, undergraduate, or graduate), and antihyperlipidemic medications (yes or no)

†Model 3 adjusted for sex (men or women), age (year), smoking (yes or no), physical activity level (low or high), education (illiterate, undergraduate, or graduate), antihyperlipidemic medications (yes or no), and history of type 2 diabetes (yes or no), hypertension (yes or no), CVD (yes or no) and cancer (yes or no)

^*^P-value < 0.05

^⁑^P-value < 0.0001

When evaluating cardiovascular mortality, the moderate-increase (HR: 1.88; 95% CI: 1.19–2.97) and the high-increase (HR: 2.82; 95% CI: 1.20–6.61) in CUN-BAE index trajectories were associated with a higher risk of cardiovascular mortality in comparison with the low-increase in CUN-BAE index (Model 2, Table 5); however, after adjusting for history of type 2 diabetes, hypertension, CVD, and cancer, the association in the high-increase in the CUN-BAE index disappeared (Model 3, Table 5). Similarly, trajectories in BRI were positively associated with the risk of cardiovascular mortality in Model 2. But after adjusting for all potential confounders, the association remains unchanged in the high-increase in BRI (HR: 3.25; 95% CI: 1.85–5.69) compared with the low-increase (Model 3, Table 5). The trajectory in ABSI and VAI was not related to the risk of cardiovascular mortality. The C-indexes of 0.883 (95% CI: 0.855–0.912) for CUN-BAE index, 0.890 (95% CI: 0.863–0.917) for BRI, 0.881 (95% CI: 0.850–0.911) for ABSI, and 0.881 (95% CI: 0.850–0.910) for VAI were found. The PH assumption was not violated for the CUN-BAE index (*P* = 0.19), BRI (*P* = 0.32), ABSI (*P* = 0.16), and VAI (*P* = 0.15).

**Table 5:** Hazard ratio (95% CI) for the incidence of cardiovascular mortality by trajectories in adiposity indices

The CUN-BAE index	no/total	Crude model	Model 1⸶	Model 2⸷	Model 3†
Low-increase	35/2790	1	1	1	1
Moderate-increase	58/3071	1.50 (0.97–2.33)	1.85 (1.18–2.91)^*^	1.88 (1.19–2.97)^*^	1.64 (1.03–2.62)^*^
High-increase	51/3069	1.23 (0.78–1.94)	3.12 (1.34–7.27)^*^	2.82 (1.20–6.61)^*^	2.17 (0.91–5.13)
**Body roundness index (BRI)**		**Crude model**	**Model 1**⸶	**Model 2**⸷	**Model 3**†
Low-increase	30/4158	1	1	1	1
Moderate-increase	66/3639	2.41 (1.52–3.81)^⁑^	1.79 (1.12–2.85)^*^	1.78 (1.11–2.84)^*^	1.55 (0.96–2.50)
High-increase	48/1126	5.71 (3.53–9.23)^⁑^	4.65 (2.72–7.98)^⁑^	4.13 (2.40–7.11)^⁑^	3.25 (1.85–5.69)^⁑^
**A body shape index (ABSI)**		**Crude model**	**Model 1**⸶	**Model 2**⸷	**Model 3**†
Low-increase	6/2152	1	1	1	1
Moderate-increase	57/4757	3.75 (1.61–8.72)^*^	1.36 (0.57–3.25)	1.20 (0.50–2.86)	1.04 (0.43–2.47)
High-increase	81/2013	12.27 (5.34–28.19)^⁑^	1.49 (0.60–3.66)	1.23 (0.50–3.03)	1.00 (0.41–2.44)
**Visceral adiposity index (VAI)**		**Crude model**	**Model 1**⸶	**Model 2**⸷	**Model 3**†
Low-increase	24/2597	1	1	1	1
Moderate-increase	74/4638	1.48 (0.93–2.36)	1.31 (0.82–2.09)	1.25 (0.78-2.00)	1.08 (0.66–1.70)
High-increase	43/1758	2.20 (1.32–3.66)^*^	2.13 (1.26–3.59)^*^	1.86 (1.09–3.18)^*^	1.38 (0.79–2.39)

The Cox proportional hazards models were used

⸶Model 1 adjusted for sex (men or women) and age (year)

⸷Model 2 adjusted for sex (men or women), age (year), smoking (yes or no), physical activity level (low or high), education (illiterate, undergraduate, or graduate), and antihyperlipidemic medications (yes or no)

†Model 3 adjusted for sex (men or women), age (year), smoking (yes or no), physical activity level (low or high), education (illiterate, undergraduate, or graduate), antihyperlipidemic medications (yes or no), and history of type 2 diabetes (yes or no), hypertension (yes or no), CVD (yes or no) and cancer (yes or no)

^*^P-value < 0.05

^⁑^P-value < 0.0001

We found no relationship between each adiposity index trajectory and cancer mortality in both crude and adjusted models (Table 6). The full model demonstrated a C-index of 0.827 (95% CI: 0.786–0.867) for the CUN-BAE index, 0.823 (95% CI: 0.781–0.864) for BRI, 0.823 (95% CI: 0.781–0.864) for ABSI, and 0.821 (95% CI: 0.778–0.863) for VAI. The PH assumption was not violated for the CUN-BAE index (*P* = 0.93), BRI (*P* = 0.98), ABSI (*P* = 0.91), and VAI (*P* = 0.86).

Results of sensitivity analysis for the incidence of outcomes by trajectories in adiposity indices are Supplementary Tables 2–5. After excluding participants with type 2 diabetes, hypertension, and those under 30 years old, we found a positive association between the moderate-increase and the high-increase in all adiposity indices and the CVD risk (Supplementary Table 2). Evidence from sensitivity analyses did not indicate any additional results for all-cause and specific causes of mortality (Supplementary Tables 3–5). The results of the Fine-Gray competing risk model are presented in Table 7.

**Table 7:** The Fine-Gray competing risk model with considering non-cardiovascular mortality as competing risk events

Excluding patients with diabetes		CUN-BAE index⸶	BRI⸶	ABSI⸶	VAI⸶
Low-increase		1	1	1	1
Moderate-increase		1.36 (1.08–1.72)	1.81 (1.46–2.23)	1.98 (1.38–2.83)	1.58 (1.25–1.99)
High-increase		2.55 (1.78–3.65)	2.06 (1.58–2.68)	1.89 (1.27–2.82)	1.92 (1.49–2.48)

The Cox proportional hazards models were used

⸶Adjusted for sex (men or women), age (year), smoking (yes or no), physical activity level (low or high), education (illiterate, undergraduate, or graduate), antihyperlipidemic medications (yes or no), and history of hypertension (yes or no), CVD (yes or no) and cancer (yes or no)

^*^P-value < 0.05

ABSI: a body shape index; BRI: body roundness index; CUN-BAE index: Clínica Universidad de Navarra-body adiposity estimator index; VAI: visceral adiposity index

## Discussion

Our longitudinal study demonstrates that long-term trajectories of adiposity indices are robust predictors of CVD in an Iranian adult population. Individuals with high-increasing trajectories of these indices faced significantly elevated risks. However, their ability to predict the risk of all-cause and cardiovascular mortality differed, as the trajectory in BRI was associated with a higher risk of the latter outcomes. In this study, the positive associations between VAI and all-cause mortality, as well as VAI and the CUN-BAE index and cardiovascular deaths, are weakened after adjusting for potential confounders. This suggests that a history of chronic disease, such as CVD, diabetes, and hypertension, may play an important role in the occurrence of death.

Findings of the present cohort are consistent with a few prospective cohort studies, mostly from China, that have established a positive association between the BRI trajectory and risk of CVD, all-cause, and cardiovascular mortality. Wu et al. [28] in a cohort of 59,278 adults aged 54.8 years, demonstrated a higher risk of CVD for the moderate-stable group (HR: 1.37; 95% CI: 1.19–1.58), the moderate-high-stable group (HR: 1.64; 95% CI: 1.40–1.91), and the high-stable group (HR: 2.03; 95% CI: 1.64–2.52) than the low-stable group. They found no association between BRI and all-cause mortality, while after removing participants under 55 years old, they confirmed a direct link between the moderate-high-stable group (HR: 1.18; 95% CI: 1.04–1.35) and the high-stable group (HR: 1.38; 95% CI: 1.13–1.67) and the risk of all-cause mortality. Results of the China Health and Retirement Longitudinal Study (CHARLS) on 9,935 adults indicated that compared with the low-stable BRI, the multivariable-adjusted HR of CVD was 1.22 (95% CI: 1.09–1.37) and 1.55 (95% CI: 1.26–1.90) for the moderate-stable and the high-stable BRI, respectively [29]. Other cohorts also reported similar findings [30, 31]. Ding et al. [32] by utilizing retrospective cohort data on 71,166 participants, revealed a significant relationship between the moderate-stable and the high-stable in BRI and the risk of all-cause mortality (HR: 1.18; 95% CI: 1.13–1.24 and HR: 1.74; 95% CI: 1.66–1.82, respectively) and cardiovascular mortality (HR: 1.12; 95% CI: 1.05–1.18 and HR: 1.64; 95% CI: 1.53–1.75, respectively) compared to the low-stable group.

In this study, VAI was related to all-cause and cardiovascular mortality, and the CUN-BAE index served as a predictor of cardiovascular death. However, after adjusting for a history of chronic diseases, their associations disappeared. Sensitivity analyses also supported these findings, indicating that, aside from BRI, trajectories in other adiposity indices are not strong predictors of all-cause or cardiovascular death in our population. In contrast, a rare study deals with the link between VAI and the CUN-BAE index trajectories and the risk of mortality and CVD outcomes.

Besides, a limited population-based study has assessed the relationship between a high trajectory of ABSI and CVD risk (HR: 1.42; 95% CI: 1.13–1.78) [33]. Although the study included middle-aged to older adults, our results were in agreement with their findings. ABSI trajectories were associated with CVD incidence in our cohort; however, their predictive power for all-cause mortality was attenuated after adjusting for potential confounders. In contrast, Kosugi et al. [21] reported that high ABSI (independent of BMI) significantly predicted mortality in older Japanese adults. This discrepancy may reflect population differences (e.g., age, ethnicity) or methodological distinctions: they analyzed ABSI as a static measure, whereas our trajectory approach accounted for intra-individual variability. Kazemian et al. [33] also revealed a direct relationship between high ABSI trajectory and the risk of all-cause mortality (HR: 1.42, 95% CI: 1.05–1.91). While both our study and the latter study were conducted among the Iranian population, this inconsistency may be explained by our participants’ relatively young baseline age (> 18 years vs. >35 years), which could influence the observed effect size. This finding suggests that adiposity indices may gain importance with age, a hypothesis warranting further investigation in our cohort.

Adiposity is implicated in systemic inflammation, insulin resistance, and endothelial dysfunction—pathways plausibly linking our observed trajectories to CVD. The BRI and VAI, which incorporate WC and metabolic markers (e.g., triglyceride, HDL-C), likely capture these pathways more accurately than BMI. Wu et al. [34] emphasized that VAI integrates WC, BMI, and lipid parameters, offering a more comprehensive metabolic snapshot than BMI alone. The VAI also includes lipid profiles, which may explain its stronger association with cardiovascular mortality, as it reflects both adiposity and dyslipidemia, a known CVD risk factor [23, 35, 36]. Notably, Yuan et al. [23] found that transitioning from low to high VAI conferred significant risk, mirroring our finding that progressive adiposity (e.g., “moderate-increase” trajectories) was detrimental. However, our study advances this by demonstrating that even gradual increases in adiposity indices over time, captured via latent class modeling, are clinically meaningful. Unlike Raffield et al. [37], who found that moderate BMI increases were protective, our moderate-increase in the CUN-BAE index trajectories showed elevated CVD risk (HR: 1.36). This discrepancy may reflect population-specific differences (Iran vs. U.S.) or the distinct metabolic implications of percent body fat (CUN-BAE index) versus BMI. It is worth noting that our results align with studies linking visceral adiposity to insulin resistance and atherosclerosis. In addition, our study adds evidence from an Iranian cohort, suggesting that visceral adiposity indices may be universally predictive across ethnicities, though regional adiposity distribution differences warrant further study.

Interestingly, we found no association between adiposity trajectories and cancer mortality. Chen et al. [38] in a large cohort of 42,022 adults aged 48.9 years, indicated a positive relationship between the high-stable BRI and cancer risk over a median 11 years of follow-up. However, they did not assess the risk of cancer mortality. Besides, we found no other published cohorts that investigated the trajectory of adiposity indices and cancer death. The absence of associations in this study may be due to the relatively smaller number of cancer-related deaths in our cohort or the nearly young age of participants at the start of the study. In addition, we had insufficient data about the family history of cancer and lifestyle habits of the participants, such as alcohol consumption and dietary intake. As the factors mentioned above are important risk factors for cancer incidence and subsequent cancer mortality, our results may be affected by a lack of them. Moreover, a small number of cancer events could influence the observed findings. Alternatively, the metabolic pathways linking adiposity to cancer may differ from those driving CVD, requiring longer follow-up or larger samples to detect significant associations.

Non-communicable diseases (NCDs) are a public health problem in Iran. Accordingly, the Iranian National Committee for Prevention and Control of NCDs (INCDC) generated a national action plan coordinated with the WHO for reducing NCD implementations [39]. Results of research in Iran indicated that applying policy interventions on tobacco, salt, and physical activity and clinical interventions on CVD and diabetes caused a significant health impact from the perspective of healthy life years gained (2,371,838) and mortalities averted (54,9428), and economic benefits (IRR 542.22 trillion) over 15 years [40]. However, due to limited resources, the healthcare workforce, and infrastructure, the challenge has persisted [39]. Our findings underscore the critical implications of lifestyle modifications and managing adiposity indices for the prevention of CVD and cardiovascular mortality in adults.

Our study has several strengths, including its large, population-based longitudinal design with a median follow-up of 18.0 years, which allowed us to model dynamic adiposity trajectories rather than relying on single-point measurements to obtain more accurate associations. The use of novel adiposity indices (BRI, VAI, ABSI, and the CUN-BAE index) provided a more nuanced assessment of body composition compared to traditional BMI, and the application of latent class growth mixture modeling captured heterogeneous risk patterns within the cohort. However, the lack of direct visceral fat imaging with DXA and CT, which could affect the interpretation of adiposity indices, is one of our limitations. Adiposity indices are useful tools for estimating visceral fat but depend on anthropometric measures like WC that cannot distinguish between visceral and subcutaneous fat. Consequently, compared to direct imaging methods, they may misjudge abdominal fat, especially in individuals with higher subcutaneous fat and lower visceral fat. Another limitation of our study was the absence of data on participants’ alcohol intake and dietary habits. Both are risk factors for adiposity and CVD and could influence our results. Although our study is population-based, demographic, cultural, and ethnic differences may limit how applicable our findings are to other populations. Additionally, while we adjusted for key confounders, the observational nature of the study precludes causal inference.

## Conclusion

Our findings highlight the importance of early and sustained weight management, as progressive increases in visceral adiposity indices such as BRI significantly elevate CVD and mortality risk. Early identification and sustained management of individuals with increasing visceral adiposity could be crucial for reducing the burden of CVD and premature mortality. Future research should focus on elucidating the biological mechanisms linking adiposity progression to adverse outcomes and developing targeted interventions to mitigate these risks.

## Electronic Supplementary Material

Below is the link to the electronic supplementary material.

Supplementary Material 1

Supplementary Material 2

## Data Availability

Not applicable.
