# Supplementary material for: Trajectories of adiposity indices and the risk of cardiovascular disease and mortality: a prospective cohort study
Source: J Transl Med. 2026 Jan 21;24:99. doi: 10.1186/s12967-025-07467-2 (PMC12822110; doi:10.1186/s12967-025-07467-2)
Supplement: Supplementary file 1 — Supplementary Material 1 [file 12967_2025_7467_MOESM1_ESM.docx]

20

25

30

35

40

45

The CUN-BAE index

0

1

2

3

4

5

Examinations

2

4

6

8

10

Body roundness index

0

1

2

3

4

5

Examinations

0.70

0.75

0.85

0.09

A body shape index

0.80

0

1

2

3

4

5

Examinations

0

2

4

6

8

Visceral adiposity index

0

1

2

3

4

5

Examinations

Supplementary Figure 1. Trajectories of adiposity indices from baseline to 2018. ( Low-increase; Moderate-increase; and High-increase in each adiposity index)
