# Supplementary material for: Trajectories of adiposity indices and the risk of cardiovascular disease and mortality: a prospective cohort study
Source: J Transl Med. 2026 Jan 21;24:99. doi: 10.1186/s12967-025-07467-2 (PMC12822110; doi:10.1186/s12967-025-07467-2)
Supplement: Supplementary file 2 — Supplementary Material 2 [file 12967_2025_7467_MOESM2_ESM.docx]

Supplementary Table 1. Formula for calculating the adiposity index

| Adiposity index | Calculation formula |
| --- | --- |
| CUN-BAE index | -44.988 + (0.503 × age) + (10.689 × sex) + (3.172 × BMI) - (0.026 × BMI^2^) + (0.181 × BMI × sex) - (0.02 × BMI × age) - (0.005 × BMI^2^ × sex) + (0.00021 × BMI^2^ × age) |
| VAI for women | [WC / (36.58 + 1.89 × BMI)] × [(TG × 0.01129) / 0.81] × [(1.52 / (HDL × 0.02586)] |
| VAI for men | [WC / (39.68 + 1.88 × BMI)] × [(TG × 0.01129) / 1.03] × [(1.31 / (HDL × 0.02586)] |
| BRI | 364.2–365.5 × √1 - [(WC / 2ℼ)^2^ / (0.5 × height)^2^] |
| ABSI | WC / (BMI^2/3^ × height^1/2^) |

Age is expressed in years.

Sex is defined as 0 for men and 1 for women.

TG and HDL are expressed in mmol/L.

WC and height are expressed in m, but for the calculation of VAI, WC is expressed in cm.

ABSI: a body shape index; BRI: body roundness index; CUN-BAE index: Clínica Universidad de Navarra-body adiposity estimator index; VAI: visceral adiposity index.

Supplementary Table 2. Sensitivity analysis for the incidence of cardiovascular disease by trajectories in adiposity indices

| **Excluding patients with diabetes** |  | **CUN-BAE index**^⸶^ | **BRI**^⸶^ | **ABSI**^⸶^ | **VAI**^⸶^ |
| --- | --- | --- | --- | --- | --- |
| Low-increase | | 1 | 1 | 1 | 1 |
| Moderate-increase | | 1.48 (1.18–1.86)^*^ | 1.84 (1.48–2.28)^⁑^ | 2.14 (1.44–3.18)^⁑^ | 1.66 (1.30–2.13)^⁑^ |
| High-increase | | 2.80 (1.85–4.22)^⁑^ | 2.19 (1.63–2.95)^⁑^ | 1.90 (1.24–2.92)^*^ | 2.11 (1.59–2.80)^⁑^ |
| **Excluding individuals under 30 years old** | | **CUN-BAE index**^†^ | **BRI**^⸷^ | **ABSI**^⸷^ | **VAI**^⸷^ |
| Low-increase | | 1 | 1 | 1 | 1 |
| Moderate-increase | | 1.30 (1.05–1.61)^*^ | 1.65 (1.35–2.01)^⁑^ | 1.84 (1.28–2.66)^*^ | 1.48 (1.17–1.86)^*^ |
| High-increase | | 2.15 (1.50–3.07)^⁑^ | 1.97 (1.53–2.54)^⁑^ | 1.89 (1.29–2.79)^*^ | 1.80 (1.39–2.34)^⁑^ |
| **Excluding patients with hypertension** | | **CUN-BAE index**^†^ | **BRI**^†^ | **ABSI**^†^ | **VAI**^†^ |
| Low-increase | | 1 | 1 | 1 | 1 |
| Moderate-increase | | 1.60 (1.24–2.04)^⁑^ | 1.82 (1.45–2.29)^⁑^ | 2.12 (1.38–3.25)^*^ | 2.13 (1.59–2.85)^⁑^ |
| High-increase | | 3.16 (2.00-4.99)^⁑^ | 2.09 (1.50–2.92)^⁑^ | 2.26 (1.42–3.61)^*^ | 2.40 (1.72–3.36)^⁑^ |

The Cox proportional hazards models were used.

^⸶^Adjusted for sex (men or women), age (year), smoking (yes or no), physical activity level (low or high), education (illiterate, undergraduate, or graduate), antihyperlipidemic medications, and history of hypertension (yes or no).

^⸷^Adjusted for sex (men or women), age (year), smoking (yes or no), physical activity level (low or high), education (illiterate, undergraduate, or graduate), antihyperlipidemic medications, and history of type 2 diabetes (yes or no), and hypertension (yes or no).

^†^Adjusted for sex (men or women), age (year), smoking (yes or no), physical activity level (low or high), education (illiterate, undergraduate, or graduate), antihyperlipidemic medications, and history of type 2 diabetes (yes or no).

^*^P-value < 0.05.

^⁑^P-value < 0.0001.

ABSI: a body shape index; BRI: body roundness index; CUN-BAE index: Clínica Universidad de Navarra-body adiposity estimator index; VAI: visceral adiposity index.

Supplementary Table 3. Sensitivity analysis for the incidence of all-cause mortality by trajectories in adiposity indices

| **Excluding patients with diabetes** |  | **CUN-BAE index**^⸶^ | **BRI**^⸶^ | **ABSI**^⸶^ | **VAI**^⸶^ |
| --- | --- | --- | --- | --- | --- |
| Low-increase | | 1 | 1 | 1 | 1 |
| Moderate-increase | | 1.17 (0.89–1.56) | 0.98 (0.76–1.27) | 1.19 (0.70–2.04) | 1.14 (0.87–1.49) |
| High-increase | | 1.05 (0.62–1.80) | 1.38 (0.97–1.97) | 1.14 (0.66-2.00) | 1.31 (0.93–1.84) |
| **Excluding individuals under 30 years old** | | **CUN-BAE index**^†^ | **BRI**^⸷^ | **ABSI**^⸷^ | **VAI**^⸷^ |
| Low-increase | | 1 | 1 | 1 | 1 |
| Moderate-increase | | 1.07 (0.84–1.37) | 1.02 (0.81–1.28) | 1.47 (0.83–2.61) | 1.18 (0.93–1.52) |
| High-increase | | 0.90 (0.58–1.39) | 1.48 (1.10–1.97)^*^ | 1.61 (0.90–2.87) | 1.27 (0.94–1.71) |
| **Excluding patients with hypertension** | | **CUN-BAE index**^†^ | **BRI**^†^ | **ABSI**^†^ | **VAI**^†^ |
| Low-increase | | 1 | 1 | 1 | 1 |
| Moderate-increase | | 0.97 (0.70–1.34) | 0.85 (0.63–1.13) | 1.12 (0.61–2.04) | 1.22 (0.89–1.67) |
| High-increase | | 0.76 (0.42–1.37) | 1.32 (0.86–2.01) | 1.25 (0.67–2.36) | 1.28 (0.84–1.93) |

The Cox proportional hazards models were used.

^⸶^Adjusted for sex (men or women), age (year), smoking (yes or no), physical activity level (low or high), education (illiterate, undergraduate, or graduate), antihyperlipidemic medications, and history of hypertension (yes or no), CVD (yes or no), and cancer (yes or no).

^⸷^Adjusted for sex (men or women), age (year), smoking (yes or no), physical activity level (low or high), education (illiterate, undergraduate, or graduate), antihyperlipidemic medications, and history of type 2 diabetes (yes or no), hypertension (yes or no), CVD (yes or no) and cancer (yes or no).

^†^Adjusted for sex (men or women), age (year), smoking (yes or no), physical activity level (low or high), education (illiterate, undergraduate, or graduate), antihyperlipidemic medications, and history of type 2 diabetes (yes or no), CVD (yes or no), and cancer (yes or no).

^*^P-value < 0.05.

ABSI: a body shape index; BRI: body roundness index; CUN-BAE index: Clínica Universidad de Navarra-body adiposity estimator index; VAI: visceral adiposity index.

Supplementary Table 4. Sensitivity analysis for the incidence of cardiovascular mortality by trajectories in adiposity indices

| **Excluding patients with diabetes** |  | **CUN-BAE index**^⸶^ | **BRI**^⸶^ | **ABSI**^⸶^ | **VAI**^⸶^ |
| --- | --- | --- | --- | --- | --- |
| Low-increase | | 1 | 1 | 1 | 1 |
| Moderate-increase | | 1.66 (0.95–2.93) | 1.86 (1.04–3.34)^*^ | 1.20 (0.41–3.51) | 0.86 (0.50–1.49) |
| High-increase | | 5.03 (1.62–15.65)^*^ | 3.70 (1.77–7.75)^*^ | 1.05 (0.34–3.25) | 1.31 (0.68–2.51) |
| **Excluding individuals under 30 years old** | | **CUN-BAE index**^†^ | **BRI**^⸷^ | **ABSI**^⸷^ | **VAI**^⸷^ |
| Low-increase | | 1 | 1 | 1 | 1 |
| Moderate-increase | | 1.60 (1.00-2.57) | 1.52 (0.93–2.46) | 1.04 (0.41–2.65) | 1.00 (0.62–1.62) |
| High-increase | | 1.98 (0.81–4.82) | 2.99 (1.69–5.29)^⁑^ | 0.96 (0.37–2.50) | 1.32 (0.75–2.31) |
| **Excluding patients with hypertension** | | **CUN-BAE index**^†^ | **BRI**^†^ | **ABSI**^†^ | **VAI**^†^ |
| Low-increase | | 1 | 1 | 1 | 1 |
| Moderate-increase | | 2.26 (1.20–4.27)^*^ | 1.69 (0.90–3.19) | 1.10 (0.31–3.86) | 1.49 (0.73–3.07) |
| High-increase | | 8.40 (1.87–37.76)^*^ | 5.08 (2.16–11.98)^⁑^ | 1.48 (0.39–5.66) | 1.60 (0.66–3.88) |

The Cox proportional hazards models were used.

^⸶^Adjusted for sex (men or women), age (year), smoking (yes or no), physical activity level (low or high), education (illiterate, undergraduate, or graduate), antihyperlipidemic medications, and history of hypertension (yes or no), CVD (yes or no), and cancer (yes or no).

^⸷^Adjusted for sex (men or women), age (year), smoking (yes or no), physical activity level (low or high), education (illiterate, undergraduate, or graduate), antihyperlipidemic medications, and history of type 2 diabetes (yes or no), hypertension (yes or no), CVD (yes or no) and cancer (yes or no).

^†^Adjusted for sex (men or women), age (year), smoking (yes or no), physical activity level (low or high), education (illiterate, undergraduate, or graduate), antihyperlipidemic medications, and history of type 2 diabetes (yes or no), CVD (yes or no), and cancer (yes or no).

^*^P-value < 0.05.

^⁑^P-value < 0.0001.

ABSI: a body shape index; BRI: body roundness index; CUN-BAE index: Clínica Universidad de Navarra-body adiposity estimator index; VAI: visceral adiposity index.

Supplementary Table 5. Sensitivity analysis for the incidence of cancer mortality by trajectories in adiposity indices

| **Excluding patients with diabetes** |  | **CUN-BAE index**^⸶^ | **BRI**^⸶^ | **ABSI**^⸶^ | **VAI**^⸶^ |
| --- | --- | --- | --- | --- | --- |
| Low-increase | | 1 | 1 | 1 | 1 |
| Moderate-increase | | 112 (0.67–1.86) | 0.77 (0.49–1.23) | 1.09 (0.45–2.66) | 1.08 (0.63–1.84) |
| High-increase | | 1.53 (0.83–2.81) | 0.80 (0.41–1.57) | 1.14 (0.45–2.94) | 0.52 (0.22–1.24) |
| **Excluding individuals under 30 years old** | | **CUN-BAE index**^†^ | **BRI**^⸷^ | **ABSI**^⸷^ | **VAI**^⸷^ |
| Low-increase | | 1 | 1 | 1 | 1 |
| Moderate-increase | | 1.42 (0.85–2.36) | 0.83 (0.53–1.30) | 1.91 (0.58–6.29) | 1.06 (0.64–1.76) |
| High-increase | | 1.46 (0.78–2.76) | 0.98 (0.52–1.84) | 1.99 (0.59–6.74) | 0.60 (0.25–1.43) |
| **Excluding patients with hypertension** | | **CUN-BAE index**^†^ | **BRI**^†^ | **ABSI**^†^ | **VAI**^†^ |
| Low-increase | | 1 | 1 | 1 | 1 |
| Moderate-increase | | 0.69 (0.35–1.36) | 0.43 (0.24–0.78)^*^ | 1.00 (0.37–2.72) | 1.27 (0.71–2.30) |
| High-increase | | 0.26 (0.09–0.72)^*^ | 0.78 (0.35–1.71) | 0.98 (0.33–2.86) | 1.11 (0.49–2.51) |

The Cox proportional hazards models were used.

^⸶^Adjusted for sex (men or women), age (year), smoking (yes or no), physical activity level (low or high), education (illiterate, undergraduate, or graduate), antihyperlipidemic medications, and history of hypertension (yes or no), CVD (yes or no), and cancer (yes or no).

^⸷^Adjusted for sex (men or women), age (year), smoking (yes or no), physical activity level (low or high), education (illiterate, undergraduate, or graduate), antihyperlipidemic medications, and history of type 2 diabetes (yes or no), hypertension (yes or no), CVD (yes or no) and cancer (yes or no).

^†^Adjusted for sex (men or women), age (year), smoking (yes or no), physical activity level (low or high), education (illiterate, undergraduate, or graduate), antihyperlipidemic medications, and history of type 2 diabetes (yes or no), CVD (yes or no), and cancer (yes or no).

^*^P-value < 0.05.

ABSI: a body shape index; BRI: body roundness index; CUN-BAE index: Clínica Universidad de Navarra-body adiposity estimator index; VAI: visceral adiposity index.
